# Lexical orthography acquisition: Is handwriting better than spelling aloud?

**DOI:** 10.3389/fpsyg.2014.00056

**Published:** 2014-02-10

**Authors:** Marie-Line Bosse, Nathalie Chaves, Sylviane Valdois

**Affiliations:** ^1^Laboratoire de Psychologie et Neuro-Cognition, Université Grenoble AlpesGrenoble, France; ^2^Laboratoire Octogone-ECCD, Université Toulouse II le MirailToulouse, France; ^3^CNRS, LPNC UMR 5105Grenoble, France

**Keywords:** orthographic acquisition, self-teaching, spelling, handwriting

## Abstract

Lexical orthography acquisition is currently described as the building of links between the visual forms and the auditory forms of whole words. However, a growing body of data suggests that a motor component could further be involved in orthographic acquisition. A few studies support the idea that reading plus handwriting is a better lexical orthographic learning situation than reading alone. However, these studies did not explore which of the cognitive processes involved in handwriting enhanced lexical orthographic acquisition. Some findings suggest that the specific movements memorized when learning to write may participate in the establishment of orthographic representations in memory. The aim of the present study was to assess this hypothesis using handwriting and spelling aloud as two learning conditions. In two experiments, fifth graders were asked to read complex pseudo-words embedded in short sentences. Immediately after reading, participants had to recall the pseudo-words' spellings either by spelling them aloud or by handwriting them down. One week later, orthographic acquisition was tested using two post-tests: a pseudo-word production task (spelling by hand in Experiment 1 or spelling aloud in Experiment 2) and a pseudo-word recognition task. Results showed no significant difference in pseudo-word recognition between the two learning conditions. In the pseudo-word production task, orthography learning improved when the learning and post-test conditions were similar, thus showing a massive encoding-retrieval match effect in the two experiments. However, a mixed model analysis of the pseudo-word production results revealed a significant learning condition effect which remained after control of the encoding-retrieval match effect. This later finding suggests that orthography learning is more efficient when mediated by handwriting than by spelling aloud, whatever the post-test production task.

## INTRODUCTION

The acquisition of word-specific orthographic knowledge is necessary to become both a fluent and efficient reader and a good speller, at least in non-transparent languages like French or English. Typically, the acquisition of specific orthographic knowledge is viewed as depending on the establishment of associations between the written and spoken forms of whole-words (Stanovich, 1993; Share, 1995; Ehri, 2005). Many studies have described the conditions for successful word-specific orthographic acquisition (see Castles and Nation, 2006, for a review). Other studies focused on the cognitive profiles of individuals with developmental dyslexia who exhibited severe problems of word-specific orthographic knowledge acquisition (Di Betta and Romani, 2006; Dubois et al., 2007). Correlational studies allowed identifying the factors related to specific orthographic knowledge (e.g., Vaessen and Blomert, 2013). However, few studies have investigated the central question of exactly how children acquire word-specific orthographic knowledge. According to the self-teaching hypothesis (e.g., Share, 1995, 1999), the building up of links between visuo-orthographic and phonological information accumulates largely via the process of successful decoding. The studies which have investigated specific orthographic learning during implicit self-teaching (Share, 1999; Cunningham et al., 2002; Share, 2004; Bowey and Muller, 2005; Cunningham, 2006; Kyte and Johnson, 2006; Martin-Chang et al., 2007; Nation et al., 2007) provided evidence for the central aspects of the self-teaching hypothesis, in showing a relation between phonological recoding skills and specific orthographic learning. Moreover, orthographic learning was found to be attenuated in conditions designed to minimize phonological recoding (Share, 1999; Kyte and Johnson, 2006), thus providing further support for the view that phonological recoding is critical to the acquisition of word-specific orthographic knowledge, as proposed by the self-teaching hypothesis.

However, phonological recoding cannot be viewed as the only cognitive factor involved in word-specific orthographic learning (e.g., Nation et al., 2007). Share (2004) reported that Hebrew-learning first graders, despite adequate decoding skills, exhibited no evidence of orthographic learning after several exposures to new words (see however, Cunningham, 2006). Cases of dyslexia with poor orthographic knowledge despite good decoding skills have also been reported (e.g., Samuelsson et al., 2000; Valdois et al., 2003). Moreover, several studies demonstrated that factors other than decoding skills, such as prior orthographic knowledge (Cunningham et al., 2002; Cunningham, 2006; Nation et al., 2007) or simultaneous visual processing (Bosse et al., in press) also relate to word-specific orthographic learning. The present study focuses on another cognitive component which may be involved in lexical orthographic acquisition: the graphomotor component.

Two recent behavioral studies have shown superior word orthographic learning from spelling practice than from reading practice alone (Shahar-Yames and Share, 2008; Ouellette, 2010). Ouellette (2010) found that orthographic training was more efficient when second graders had to spell the item they just read than when they had to think silently to the item they just read. Shahar-Yames and Share (2008) showed an advantage of spelling practice in comparison with reading practice alone, even when the number of item’s exposures was the same in both practices. The first explanation of the advantage of a spelling practice is that spelling involves more processing than reading. It requires exhaustive letter-by-letter consideration and fully operational connections between sounds and letters (Perfetti, 1997). Moreover, spelling involves higher processing demands (Bosman and van Orden, 1997). Furthermore, when spelling a word several times, the child must process each and every letter exhaustively at each spelling. On the contrary, when reading a word several times, the representation may be less than fully specified yet sufficient for word identification (Holmes and Carruthers, 1998). Finally, as the spelling practice is generally associated with immediate recall (i.e., the word spelled is not visible when the child spell it), the advantage of spelling practice could be explained by processing in working memory during immediate recall.

Another specific aspect of spelling practice likely to enhance lexical orthographic acquisition concerns the motor-kinesthetic aspects of spelling production. In classrooms, children learn concomitantly to read and write. Each letter is associated with highly specific writing movements and each word letter-string with specific graphomotor patterns. Accordingly and in line with the embodied cognition theory (Barsalou et al., 2003; Shapiro, 2010) which proposed that all cognitive operations are embedded in the current state of the body and sensorimotor brain systems, graphomotor patterns and visual orthographic features may form a sensory-motor representation of orthographic knowledge in memory. There is already strong evidence from behavioral and neuroimaging data that writing movements are involved in letter memorization. In young children, handwriting contributes more than typing to the visual recognition of isolated letters (Longcamp et al., 2005). Moreover, the visual presentation of a single letter activates the premotor regions involved in handwriting, even when the task does not require any motor response (e.g., Longcamp et al., 2008). Interestingly, the lateralization of this premotor activation depends on the participant’s writing hand. The left premotor cortex is activated in right-handed individuals, the right premotor cortex in left-handed. Such findings suggest a reactivation of motor knowledge during single letter visual processing. As for words, teaching studies showed that spelling practice boosts reading acquisition (e.g., Conrad, 2008; Ouellette and Sénéchal, 2008). However, the contribution of handwriting to reading is still debated (e.g., Tan et al., 2005; Bi et al., 2008, for a debate concerning Chinese language). Moreover, the role of handwriting on lexical orthographic memorization, that is memorization of letter strings, has not been clearly established yet.

To summarize, two causal hypotheses are generally proposed to explain why word spelling practice improves lexical orthographic learning more than word reading practice alone. The first hypothesis is that spelling involves more exhaustive letter-by-letter processing; the second one is that spelling provides the additional motor-kinesthetic information that contributes to the word representation in memory. However, the behavioral studies that concluded to an advantage of the word spelling practice did not differenciate between these two causal hypotheses (Shahar-Yames and Share, 2008; Ouellette, 2010). Indeed in the experiments they reported, the compared learning practices differed on at least two features: processing demand (immediate recall only during the spelling practice) and motor activity (handwriting only during the spelling practice). The main goal of the present study was to test whether the spelling practice advantage on lexical orthographic learning is mainly due to exhaustive letter-by-letter processing, or whether additional motor-kinesthetic information yields more efficient learning. For this purpose, a spelling-by-hand practice was compared with a spelling-aloud practice. Both practices involved exhaustive letter-by-letter processing and immediate recall, but only the spelling-by-hand practice provided additional motor-kinesthetic information. In line with the embodied cognition statement that motor information and visual perception both contribute to form a unified representation of objects, we expected the spelling-by-hand practice to favor orthographic memorization more than the spelling aloud practice.

## EXPERIMENT 1

### METHOD

#### Participants

Twenty French children participated in the experiment. They were recruited from four classes in two public schools of Guadeloupe (France). Their mean chronological age was 10 years 5 months (SD = 4 months; range = 9 years 8 months–11 years) for a mean reading age (Lefavrais, 1965) of 10 years 4 months (SD = 9 months, range = 9 years 1 month–11 years 10 months), thus showing normal reading acquisition. They further exhibited on the average mental reasoning abilities with a mean raw score of 37.8 (SD = 7; range = 22–49) on the Raven Standard Progressive Matrices (Raven et al., 1998). Informed written consent was obtained from both the participants and their parents.

#### Material

During the learning phase, participants were asked to read and immediately recall 12 bisyllabic pseudowords (PW) that were embedded in short sentences. During the test phase, they were subsequently asked to retrieve or recall the target PWs’ orthographic form. Each pseudo-word was made of existing French trigrams (see **Table 1**) to build-up a legal pronounceable orthographic sequence. Each contained at least two inconsistent phonemes that could be translated using different graphemes. For example, the pseudo-word /litN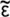/ was associated with the written form “LITAIN” during the learning phase, but spellings as “LITEIN” or “LITTIN” are other plausible ways of transcoding this same phonological pseudo-word in French. Target spellings never corresponded to the orthographic form generated through application of the most frequent phoneme-grapheme correspondences (“LITIN” in the previous example). A target inconsistent phoneme was always present in two paired pseudo-words, each time associated with a different grapheme (e.g., the phoneme /f/ is spelled “F” in “fenval” – /fãval/, but “PH” in “phoril” – /foRil/). Two sets of six pseudo-words, composed of one member of the pair each, were designed. The two sets were matched for trigram frequency. Each child was exposed to the two sets of matched pseudo-words (for instance including the /f/ – “PH” and /f/ – “F” correspondence) in different learning sessions, so that systematic application of a phoneme–grapheme correspondence (e.g., /f/→“PH”) during the test phase would yield errors. The two sets of pseudo-words are listed on **Table 1**. Following the self-teaching paradigm, each pseudo-word was embedded into two short sentences as in the following example:

**Table 1:** Pseudo-word sets (inconsistent phoneme-grapheme correspondences in bold).

Set 1	Set 2
li**tain**	**qu**ota**rt**
v**ai**ga**rd**	l**y**on**in**
**fen**val	a**llan**de
m**i**st**ond**	la**ttont**
m**aul**an	**pho**ril
**c**arv**ie**	t**ei**n**it**

“Dans un pays lointain, un magicien fabrique un bonbon qui s’appelle un **mistond**. Il a des pouvoirs magiques; si tu manges un **mistond** tu deviens invisible” (In a distant country, a magician makes a candy, called a mistond. It has magic powers. If you eat a mistond you become invisible).

The semantic context was different for each item and inspired from the texts used by Share (1999) in his first self-teaching experiment. Sentences contained 14 words on average. All words but the target items were frequent words.

#### Learning phase: two conditions of learning

Participants were engaged in two “learning” conditions of handwriting and spelling aloud. Half pseudo-words in each set were processed following the handwriting condition, the other half following the spelling aloud condition. Pseudo-word sets were fully counterbalanced across learning conditions.

– In the *handwriting condition*, children were asked to read aloud the text composed of the two short sentences, so that each target PW was decoded twice. Immediately after each text reading, the printed text was removed. The examiner provided the oral pronunciation of the target PW to the child who was asked to handwrite the target PW from memory (immediate recall). The child’s first spelling was then removed and he was asked to write the target PW again from memory. No feedback was given. In this learning condition, the child visually processed each target item four times: twice during text reading and twice during handwriting. This handwriting learning condition was designed from Shahar-Yames and Share (2008).
– In the *spelling aloud condition*, participants were asked to read the two short sentences twice each, so that each child was exposed four times to the orthographic form of the target PW while reading. Immediately after reading, the printed text was removed. The target PW was spoken aloud by the experimenter and participants were asked to spell the PW aloud twice (immediate recall). No feedback was given. In this learning condition as in the previous one, the child visually processed each target item four times during text reading.
  The two learning conditions were carefully equated except for the handwriting component. First, spelling production was done from memory to control for the number of item’s visual exposures. In both conditions, children were asked to recall the item twice from memory (immediate recall) and they were visually exposed four times to each item. Note that this short training is typically considered as sufficient for orthographic learning. Previous self-teaching studies have demonstrated that learning took place even after a single exposure (Share, 2004; Nation et al., 2007). Target PW reading performance and the spelling aloud response were recorded (as was the handwriting response) to later control for any difference between the learning conditions.

#### Test phase: two tasks to assess orthographic learning

One week after the learning phase, orthographic learning of the target PWs was assessed through tasks of spelling to dictation and orthographic choice. The learning phase and the test phase were separated by 1 week, to assess long-term memory of the new orthographic string.

– The *spelling to dictation task* required the participants to write the target PWs immediately after their pronunciation by the experimenter. The experimenter specified that the dictated PWs were those introduced 1 week earlier in the two sentences that the child had been asked to read. The child was asked to write these PWs just as they were spelled in the sentences. The target PWs of each set were dictated in a random order. We scored 1 point for each accurately spelled PW.
– In the *orthographic choice task*, four homophone heterographs of the target PWs were presented including the learned orthographic sequence. The homophones were written on a single line and the position of the target PW was varied at random (e.g., fauril phauril foril **phoril**). Participants had to put a circle around the target orthographic pseudo-word.

#### Design

This study was a within-participant design with two conditions of learning (handwriting and spelling-aloud). Each participant had to learn the orthographic form of all 12 PWs, half processed in the handwriting learning condition, the other half in the spelling-aloud learning condition. Items were counterbalanced across conditions. The study was carried out over three individual sessions separated by an interval of 1 week. Children were assessed individually in a quiet room of their school. Session 1 began with the Reading Test (Lefavrais, 1965) used to estimate the child’s reading age, followed by the learning phase of 6 target PWs (set 1 or set 2 with 3 PWs in each learning condition). One week later, Session 2 began with the test phase for the 6 PWs introduced the week before, followed by the learning phase of the remaining 6 PWs (set 2 or set 1, 3 in each learning condition). One week later, Session 3 was devoted to the test phase for the 6 target PWs introduced the week before. The Raven Matrices test (Raven et al., 1998) was administrated at least 1 week before Session 1, collectively in the classroom.

#### Rationale and hypotheses

The rationale for the study was as follows. We reasoned that if new words’ orthographic learning mainly relies on accurate processing of their orthographic sequence during reading and on exhaustive letter-by-letter processing during immediate recall, then participants should exhibit similar orthographic learning in both the spelling-aloud learning condition and the handwriting learning condition (four PWs’ occurrences and two immediate recalls in both conditions). This prediction should be verified provided that decoding and immediate recall were as accurate in one condition as the other. Now if the association of a motor program contributes to orthographic learning, then the orthographic form of the target PWs should be more efficiently learned in the handwriting condition than in the spelling-aloud condition. Better orthographic learning was expected to result in higher performance in both the spelling under dictation task and the orthographic choice task. As spelling to dictation relies on the accurate memorization of the whole PW letter string, a higher performance was expected in the orthographic choice task (based on PW recognition).

### RESULTS

#### Preliminary analyses: pseudo-word processing in the learning phase

Analyses were first carried out to assess whether pseudo-word reading accuracy and pseudo-word orthography immediate recall differed in the two learning conditions. The rate of PWs accurately read and accurately recalled is provided in **Table 2** for the two learning conditions.

**Table 2:** Experiment 1. Mean target reading accuracy and mean accurate orthography immediate recall (Percentage and standard deviation) during the handwriting and spelling-aloud learning conditions for each session.

	Learning condition	
	Handwriting	Spelling aloud
**Reading accuracy**
Session 1	100 (0)	98.7 (4.1)
Session 2	99.2 (3.7)	99.2 (2.6)
**Orthography immediate recall**
Session 1	86.7 (18.4)	93.3 (11.3)
Session 2	85 (20.9)	97.5 (8.2)

Most target PWs were accurately decoded and their orthography accurately recalled immediately after reading. For reading accuracy, there were no significant Learning Condition effect, no Session effect (both *F* < 1), and no Session by Learning Condition interaction [*F*(1,19) = 1.3, ns]. However, a significant Learning Condition effect [*F*(1,19) = 12.4, *p* < 0.01] was found in immediate recall. PWs orthography was more accurately recalled in spelling aloud than in handwriting. There was no Session effect and no Session × Learning condition interaction (both *F* < 1). A qualitative analysis showed that recall errors included letter substitutions (39%), letter deletions (37%), and letter additions (24%) in the same proportion across conditions (respectively for handwriting and spelling aloud: 37 vs. 46% for letter substitutions, 39 vs. 27% for letter deletions, 24 vs. 27% for letter additions).

Overall, the preliminary analyses showed that items’ reading was as accurate in one learning condition as the other, and that items’ immediate recall was slightly better in the spelling aloud condition than in the handwriting condition.

#### Orthographic learning

***Spelling to dictation.*** The spelling to dictation task is a very difficult task in an opaque language like French. Indeed, each inconsistent phoneme could be spelled with several graphemes, frequently more than three (e.g., a final /R/ could be spelled r, re, rre, rt, rd, rs). Thus, our target disyllabic pseudo-words that included at least two inconsistent graphemes each could be hardly spelled accurately by chance. However, as shown in **Table 3**, children accurately spelled under dictation more than 30% of the items. An ANOVA was performed on correct responses with Learning Conditions (handwriting or spelling aloud) and Sessions (1 or 2) as repeated measures. There was a significant main effect of Learning Condition [*F*(1,19) = 7.5, *p* < 0.05], showing that the orthography of target PWs was more accurately learned in the handwriting condition than in the spelling-aloud condition. No Session effect was found [*F*(1,19) = 1.9, ns] and no significant Session × Learning Condition interaction [*F*(1,19) = 1.7, ns].

**Table 3:** Experiment 1. Mean accuracy performance (percentages and standard deviations) in the spelling to dictation and orthographic choice tasks as a function of learning conditions and sessions.

	Learning condition	
	Handwriting	Spelling aloud
**Spelling to dictation**
Session 1	45 (29)	21.7 (25)
Session 2	30 (28)	25 (28)
**Orthographic choice**
Session 1	68.3 (33)	63.3 (34)
Session 2	58.3 (32)	66.7 (24)

***Orthographic choice task.*** In the orthographic choice task, participants had to recognize the target PW among four homophone heterographs, so that 25% correct performance was at chance level. **Table 3** shows that the observed levels of performance were largely above chance in all learning conditions (64% of correct choices on average). The ANOVA performed on correct responses in the orthographic choice task revealed no significant Learning Condition or Session effect (both *F* < 1) and no interaction [*F*(1,19) = 1.1, ns].

### CONCLUSION

In Experiment 1, the ability of children to memorize the orthographic sequence of PWs was assessed following two learning conditions of handwriting and spelling aloud. The aim was to determine whether the handwriting learning condition yielded better orthographic memorization than the spelling aloud learning condition. The results suggest that the handwriting learning condition provided added benefits for spelling production (the spelling to dictation post-test) but not for spelling recognition (the orthographic choice task). This result is consistent with the idea that handwriting is more efficient than spelling aloud to memorize orthography for production (but not for recognition).

However, the significant advantage of the handwriting learning condition could also be explained by a similarity effect between the learning condition and the post-test condition. Indeed, the spelling to dictation post-test was a handwriting task. A second experiment was conducted to disentangle encoding-retrieval matching effects from specific handwriting effects. In Experiment 2, the learning phase was similar as in Experiment 1 but a spelling aloud production task was used as post-test instead of the handwriting production task. If handwriting is more efficient than spelling aloud to memorize orthography for production independently of the post-test production mode, then we should obtain the same effect as in Experiment 1.

## EXPERIMENT 2

### PARTICIPANTS

Twenty French children participated in the experiment. They were recruited from two classes in two public schools in the Gers, France. Their mean chronological age was 10 years 8 months (SD = 9 months; range = 11 years 11 months–9 years) for a mean reading age (Lefavrais, 1965) of 10 years 4 months (SD = 1 year 9 months, range = 12 years 10 months–8 years 1 month), thus showing normal reading acquisition. They further exhibited on the average mental reasoning abilities with a mean raw score of 43.2 (SD = 5.7; range = 34–54) on the Raven Standard Progressive Matrices (Raven et al., 1998). Informed written consent was obtained from both the participants and their parents.

### MATERIAL

Material and design were exactly the same as in Experiment 1, except for the first task of the post-test phase which was a spelling aloud to dictation task instead of a spelling down to dictation task.

The *spelling aloud to dictation task* required the participants to spell aloud the target PWs immediately after their pronunciation by the experimenter. The experimenter specified that the dictated PWs were those introduced 1 week earlier in the two sentences that the child had been asked to read. The child was asked to spell aloud these PWs just as they were spelled in the sentences. The experimenter immediately wrote the child’s response on a paper unseen by the participant. The target PWs of each set were dictated in a random order. We scored 1 point for each PW accurately spelled aloud.

### RESULTS

#### Preliminary analyses of pseudo-word processing in the learning phase

The rate of PWs accurately read and accurately recalled during the learning phase is provided in **Table 4**. Most target PWs were accurately decoded and their orthography accurately recalled immediately after reading. For both reading accuracy and immediate recall, there were no significant Learning Condition effect, no Session effect and no interaction (*F*s < 1). The qualitative analysis showed letter substitution (58%), letter deletion (22%), and letter addition (20%) errors which were found in similar proportion in the handwriting and spelling aloud conditions (55 vs.60% letter substitutions, respectively, 26 vs.19% letter deletions, 18 vs. 21% letter additions).

**Table 4:** Experiment 2. Mean accuracy (in percentages) and standard deviations for target reading accuracy and for target orthography recall during, the learning phase, according to conditions and sessions.

	Learning condition	
	Handwriting	Spelling aloud
**Reading accuracy**
Session 1	85.8 (19.7)	87.9 (19.4)
Session 2	88.3 (18.8)	90.8 (17.1)
**Immediate recall**
Session 1	86.7 (19.9)	82.5 (19.8)
Session 2	86.7 (15.9)	81.7 (25.3)

As in Experiment 1, the preliminary analyses showed that items’ reading was as accurate in one learning condition as in the other. Items’ immediate recall was as good in the spelling aloud learning condition than in the handwriting learning condition.

#### Post-test orthographic learning

***Spelling aloud to dictation.*** The Experiment 2 spelling aloud to dictation task was at least as difficult as the Experiment 1 spelling by hand to dictation task. Target disyllabic pseudo-words that included at least two inconsistent graphemes each could be hardly spelled aloud accurately by chance. However, as shown in **Table 5**, children accurately spelled aloud more than 22.5% of the items on average. An ANOVA was performed on correct responses with Learning Conditions (handwriting or spelling aloud) and Sessions (1 or 2) as repeated measures. There was a near to significance main effect of Learning Condition [*F*(1,19) = 4.2, *p* = 0.055] showing that the orthography of target PWs was more accurately learned in the spelling aloud learning condition than in the handwriting learning condition. There was a near to significance Session effect [*F*(1,19) = 3.1, *p* = 0.10] but no significant interaction (*F* < 1).

**Table 5:** Experiment 2. Mean accuracy (in percentages) and standard deviations for spelling aloud to dictation task and for orthographic choice task, according to conditions and sessions.

	Learning condition	
	Handwriting	Spelling aloud
**Spelling aloud to dictation**
Session 1	21.7 (25)	33.3 (31)
Session 2	13.3 (17)	21.7 (20)
**Orthographic choice**
Session 1	63.3 (28)	61.7 (29)
Session 2	51.7 (30)	41.7 (28)

***Orthographic choice task.*** In this task, participants had to recognize the target among four homophone heterographs, so that 25% correct performance was at chance level. **Table 5** shows that the observed levels of performance were largely above chance in all learning conditions (55% correct choices on average). The ANOVA performed on correct responses in the orthographic choice task revealed a non significant Learning Condition effect (*F* < 1) a significant Session effect [*F*(1,19) = 6.7, *p* < 0.05] and no significant interaction (*F* < 1).

#### Mixed model analysis

Results of Experiment 1 and Experiment 2 suggest an encoding-retrieval match effect. Indeed, in both experiments, performance in the post-test spelling production task (writing to dictation in Experiment 1 vs. spelling aloud to dictation in Experiment 2) was higher for items learned under similar conditions (handwriting in Experiment 1 vs. spelling aloud in Experiment 2) than for items learned under dissimilar conditions (spelling aloud in Experiment 1 vs. handwriting in Experiment 2). To better evaluate the relative weights of the learning condition, post-test condition and encoding-retrieval match effect on orthographic acquisition, we conducted a mixed model analysis (controlling variability between participants and between items, i.e., random effects) on the post-test spelling to dictation results of both experiments. The spelling post-test condition (writing or spelling aloud) was considered as an independent variable just as the learning condition and session. The interaction between learning condition and post-test spelling condition was also introduced in the equation as the encoding-retrieval match variable. As participants’ reading accuracy and immediate recall performance during the learning phase could vary from one item to the other, these variables were also introduced in the equation.

**Table 6** provided β values for all fixed effects with their z-values and significance. Once random factors were taken into account, Learning Condition, Post-test condition, and Session remained as significant predictors of performance in the post-test spelling task. The interaction between learning condition and post-test condition (i.e., the match effect) was also highly significant (β = 1.56, *p* < 0.001). Immediate recall performance during the learning phase was also significant but not reading accuracy.

**Table 6:** Mixed model analysis on the post-test spelling to dictation results of both experiments. β values for all fixed effects with their *z*-values and significance.

Variables	β values	*z*-Values
Spelling post-test condition	1.17	3.06**
Spelling learning condition	0.74	2.15*
Interaction learning-post-test	-1.56	-3.34***

Session	-0.48	-2.11*
Reading accuracy during learning	0.00	0.04
immediate recall performance during learning	0.01	2.5*

## DISCUSSION

The main goal of the present study was to explore whether the advantage of handwriting practice on lexical orthographic acquisition is mainly due to exhaustive letter-by-letter processing or to additional motor-kinesthetic information. Children were asked to read pseudo-words embedded in sentences and to immediately recall their orthography, either by handwriting them or by spelling them aloud. One week later, knowledge about PWs orthography was assessed through a spelling to dictation task and a recognition task. The handwriting learning condition [a replication of Shahar-Yames and Share (2008) spelling practice learning condition] was compared to a spelling-aloud learning condition. Both practice learning conditions involved exhaustive letter-by-letter processing and immediate recall but only the handwriting practice provided additional motor-kinesthetic information. If handwriting movements contribute to orthography learning, we expected the spelling-by-hand more than the spelling aloud practice to favor orthographic memorization.

The results of Experiment 1 confirmed those of previous experiments (Shahar-Yames and Share, 2008; Ouellette, 2010) showing that the handwriting learning condition provided added benefits for spelling production (the spelling to dictation post-test) but not for spelling recognition (the orthographic choice task). This finding is consistent with the idea that handwriting is more efficient than spelling aloud to memorize orthography for production (but not for recognition). As the two learning conditions involved exhaustive letter-by-letter processing, similar letter-by-letter immediate recall and an equivalent number of visual exposures to target PWs, higher efficiency of the handwriting practice on orthographic acquisition would more likely follow from the additional motor-kinesthetic information inherent to handwriting practice. It is further noteworthy that the handwriting practice advantage on long-term memorization was significant despite higher immediate recall performance for the spelling aloud learning condition.

However, when the post-test production task was a spelling aloud task instead of a spelling-by-hand task (Experiment 2), no benefit of handwriting learning was found for either the spelling production (the spelling aloud to dictation) or the spelling recognition (the orthographic choice) post-tests. To the contrary, the only benefit found was for the spelling aloud production task when preceded by the spelling aloud learning condition. This result suggests that most of the orthographic memory differential effects found between the handwriting and the spelling aloud learning conditions could be explained by an encoding-retrieval match effect. The mixed model analysis computed on Experiment 1 and Experiment 2 results confirmed a strong significant interaction between the learning conditions and the post-test production tasks. It is well known that match between encoding and retrieval conditions affects memory performance (e.g., Godden and Baddeley, 1975; Roediger and Guynn, 1996). The encoding-retrieval match principle states that memory performance is enhanced if the type of task at encoding matches the type of task at retrieval (see, however, Nairne, 2002). Then, when an item has been handwritten in the learning phase, its orthography is expected to be more accurately recalled by handwriting than by spelling aloud. Reversely, when the item was spelled aloud during the learning phase, better retrieval is expected through spelling aloud than through handwriting.

Results of the mixed model analysis further showed a significant learning condition effect. This result means that orthography learning was slightly more efficient by handwriting training than by spelling aloud training, whatever the post-test production task. The main difference between the two learning conditions was their spelling mode. The handwriting condition involved a hand movement, the spelling aloud condition involved an articulatory movement. A first interpretation of the results is to say that memorization of hand movements could support orthographic memorization more efficiently than memorization of articulatory movements. Movements made when handwriting new words could be crucial for learning, in providing additional associative links between the writing form and the oral form of the item (Pérez et al., 2012). In line with this assumption, multi-sensory teaching experiments emphasized the importance of kinesthetic information in letter or word processing (e.g., Hulme et al., 1987; Bara and Gentaz, 2011). A few studies have focused on the contribution of the handwriting grapho-motor component to child spelling, by comparing different modes of spelling. In line with the present results, they concluded that handwriting was more effective than other spelling modes. For example, Cunningham and Stanovich (1990) compared handwriting, computer keyboard typing and letter tiles manipulation. They found superior spelling outcomes when children handwrote words, compared with the two other conditions.

However, other interpretations of these results could also be made. Indeed, it is known that spelling by hand is a very familiar task, largely automated for fifth graders (Maeland and Karlsdottir, 1991). On the contrary, spelling aloud is rarely used in French schools to learn word knowledge. Then, we could hypothesize that, at least for French fifth graders, handwriting is much more familiar and automated than spelling aloud. Then, it could be simply more natural for them to learn new words by this medium. For example, writing letter strings could require less attention than spelling aloud the same letter strings, so that more attention may be devoted to high level processing (e.g., memorization) in the former task. Independently of task familiarity and automaticity, another plausible explanation is to hypothesis a specific role of visual feedback on orthographic memorization. We already know that visual feedback is useful for letter production (e.g., Vinter and Chartrel, 2010). Finally, even if the observed advantage of the handwriting training cannot be straightly attributed to the motor component, the data evidenced slightly but significantly greater performance for words trained by hand.

The mixed model analysis further revealed a significant main effect of post-test production mode, showing that items were better recalled in the spelling by hand to dictation task than in the spelling aloud to dictation task, whatever the learning task. The different explanations of the learning condition effect, discussed above, could also explain this post-test mode effect: a role of the motor component, an effect of the task familiarity or a role of visual feedback.

None of the present experiments revealed a main effect of learning condition on the post-test recognition task. Only those measures requiring spelling production revealed statistically reliable differences between the learning conditions (see Shahar-Yames and Share, 2008, for similar results). According to these results, handwriting may be especially beneficial for orthographic production, as compared to orthographic recognition. However, several studies have demonstrated that handwriting training contributes to letter recognition, in addition to letter production. Studies on kindergarten children (Longcamp et al., 2005) or adults (Longcamp et al., 2006, 2008) showed that handwriting training was more efficient than typing training to recognize letters or letter orientations (see also Longcamp et al., 2010). fMRI recordings during character recognition showed a reactivation of motor knowledge (Longcamp et al., 2008; James and Engelhardt, 2012). To explain the absence of learning condition effect on orthographic recognition in the present experiments, we suggest that, in order to be reactivated during item recognition, the grapho-motor pattern of the item has to be sufficiently learned and automated. Accordingly, a significant advantage of the handwriting learning condition on the post-test recognition task should only be observed if the learning phase includes a sufficient number of handwriting repetitions to permit gesture automation.

To conclude, the main result of this study is that a large part of the learning by handwriting practice advantage on long-term lexical orthographic production (Shahar-Yames and Share, 2008; Ouellette, 2010) is explained by an encoding-retrieval match effect. As a consequence, insofar as children are frequently asked to handwrite rather than to spell words aloud at school, the handwriting practice for learning orthography is fully justified and must be encouraged. Orthography learning was also slightly more efficient by handwriting than by spelling aloud, whatever the post-test production task, which may suggest a contribution of graphomotor information to orthographic acquisition. However, such a contribution should have resulted in a positive handwriting training effect on PW recognition, which was not found.

## Conflict of Interest Statement

The authors declare that the research was conducted in the absence of any commercial or financial relationships that could be construed as a potential conflict of interest.
